# Supplementary material for: The Effect of Noise Exposure on High-Frequency Hearing Loss among Chinese Workers: A Meta-Analysis
Source: Healthcare (Basel). 2023 Apr 10;11(8):1079. doi: 10.3390/healthcare11081079 (PMC10137611; doi:10.3390/healthcare11081079)
Supplement: Supplementary file 1 [file healthcare-11-01079-s001.zip › healthcare-2267430-supplementary.pdf]

Supplementary Materials

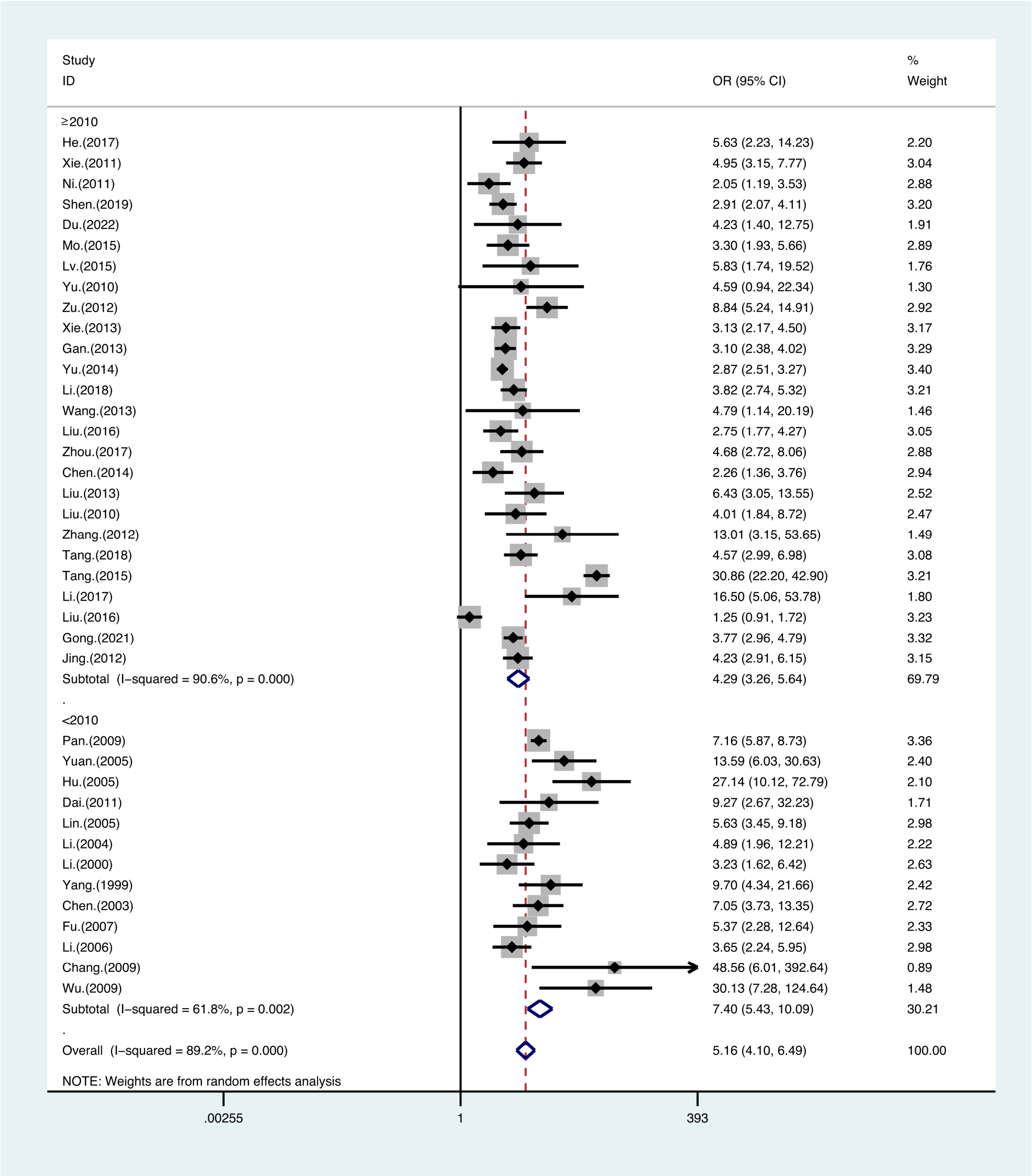

Figure S1. Forest plot for the association between publication year and HFNIHL risk.

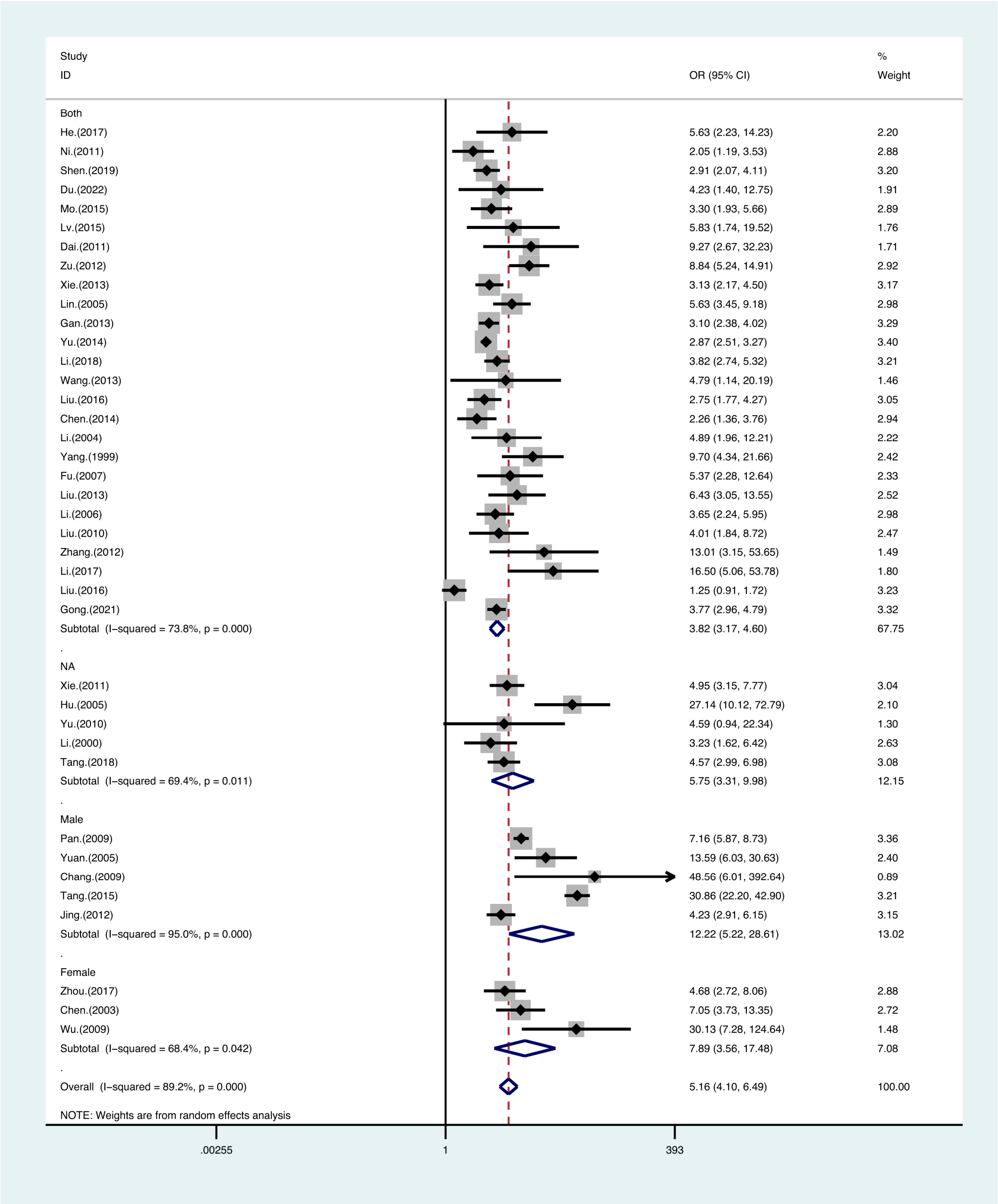

Figure S2. Forest plot for the association between gender and HFNIHL risk.

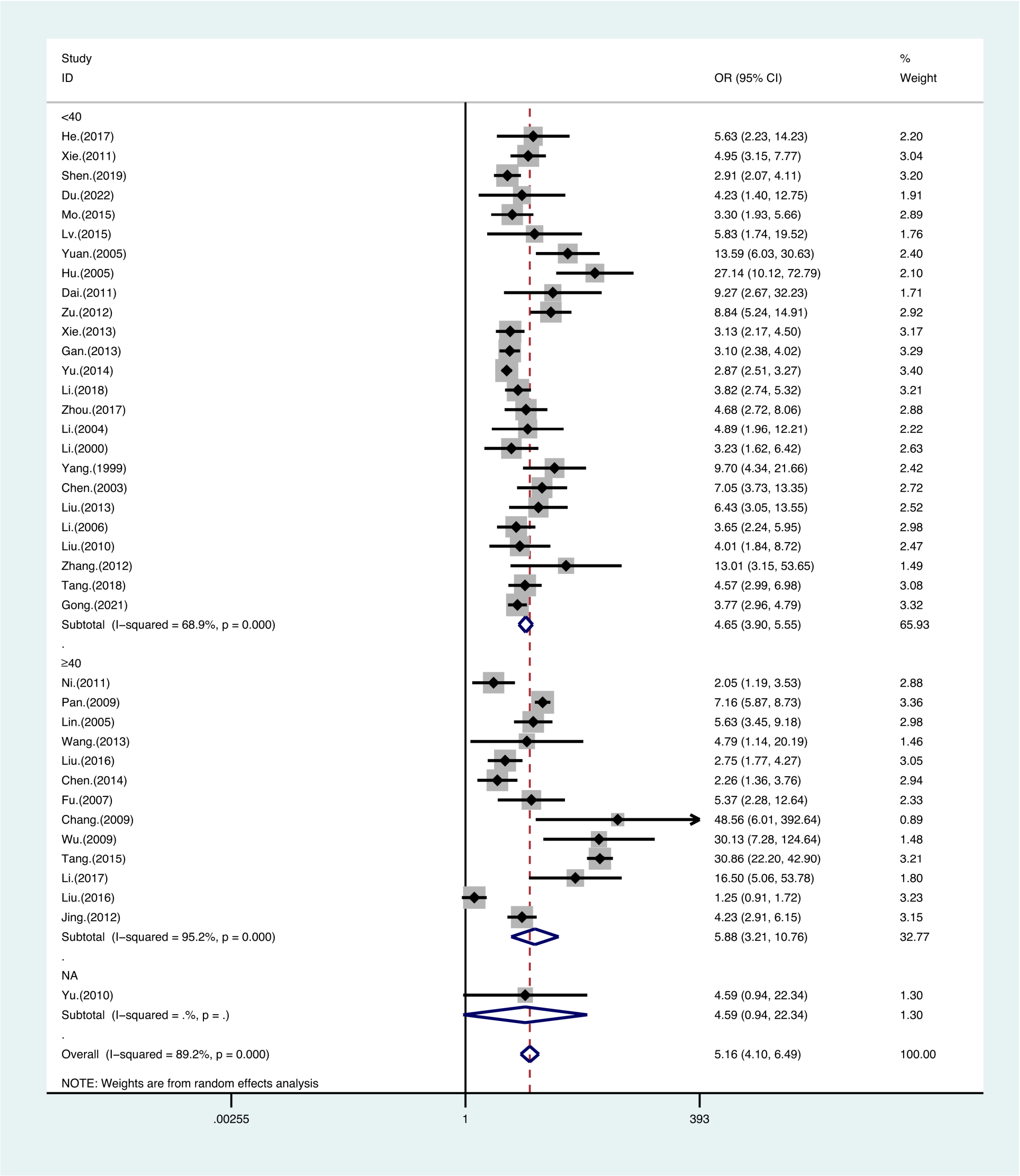

Figure S3. Forest plot for the association between age and HFNIHL risk.

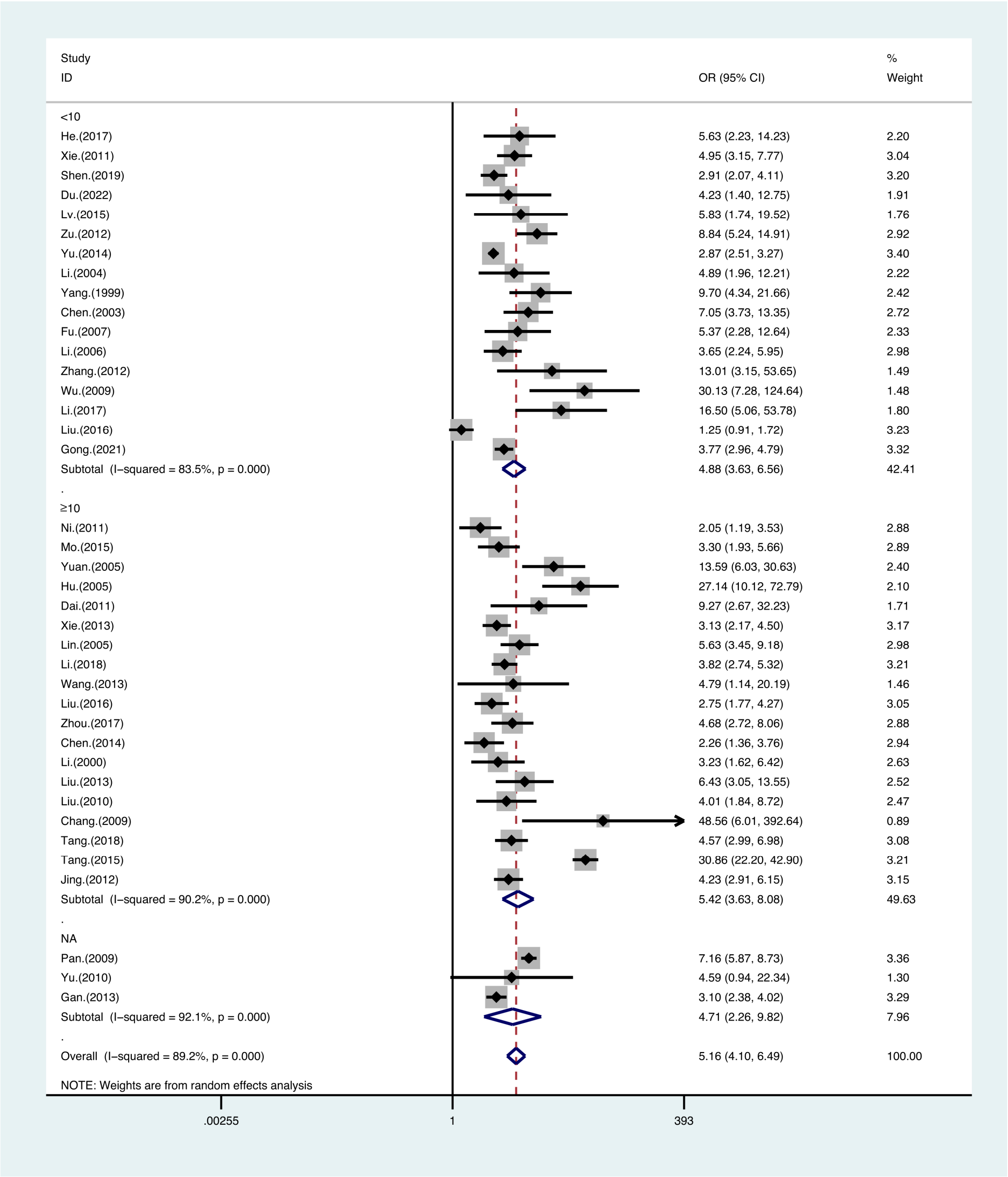

Figure S4. Forest plot for the association between length of work and HFNIHL risk.

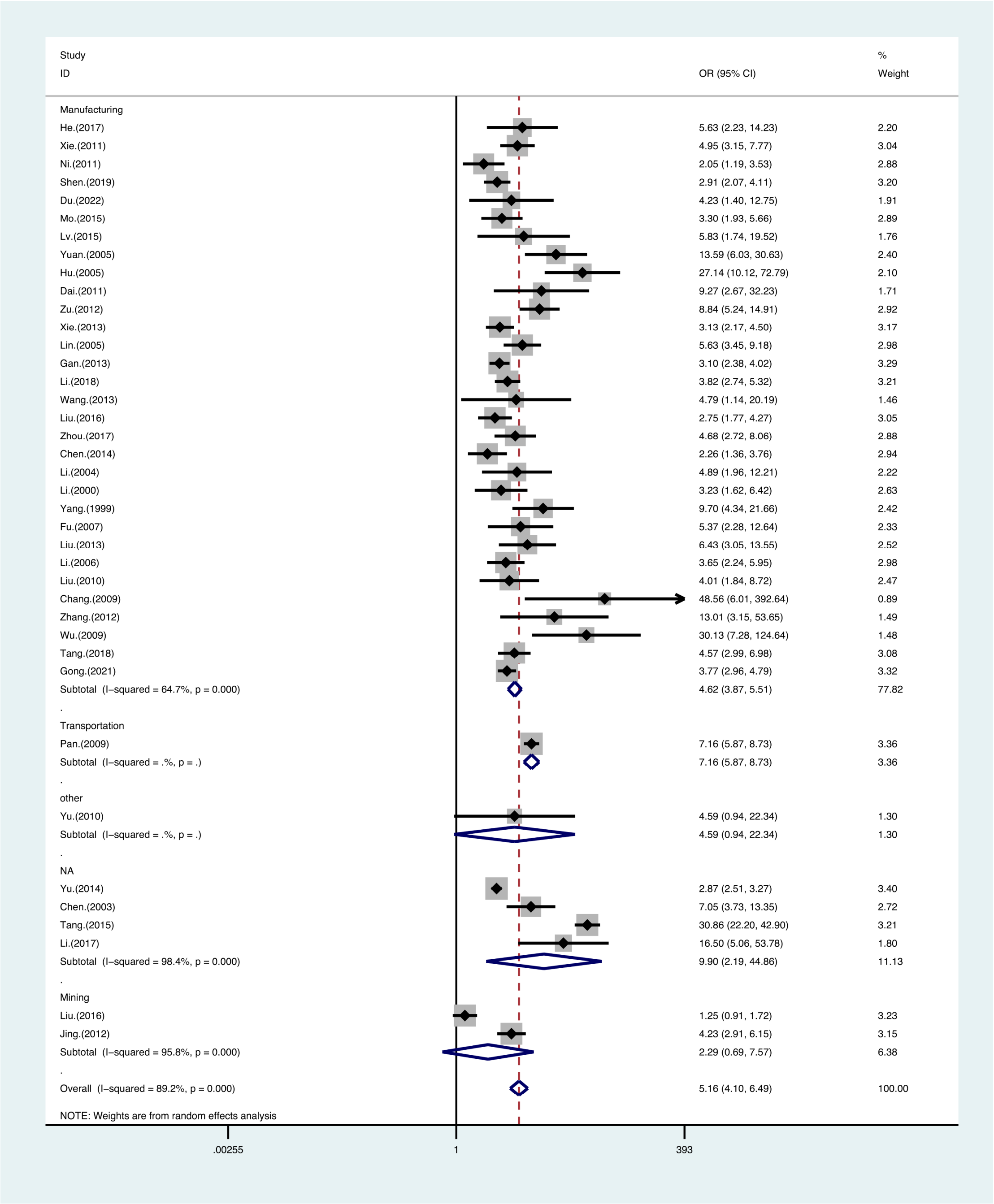

Figure S5. Forest plot for the association between type of industry and HFNIHL risk.
